# Exonization of a deep intronic long interspersed nuclear element in Becker muscular dystrophy

**DOI:** 10.3389/fgene.2022.979732

**Published:** 2022-08-25

**Authors:** Zhiying Xie, Chang Liu, Yanyu Lu, Chengyue Sun, Yilin Liu, Meng Yu, Junlong Shu, Lingchao Meng, Jianwen Deng, Wei Zhang, Zhaoxia Wang, He Lv, Yun Yuan

**Affiliations:** ^1^ Department of Neurology, Peking University First Hospital, Beijing, China; ^2^ Department of Neurology, Peking University People’s Hospital, Beijing, China; ^3^ Department of Pathology, Peking Union Medical College Hospital, Beijing, China

**Keywords:** *DMD*, LINE-1, aberrant splicing, deep-intronic structural variant, long-read sequencing

## Abstract

The precise identification of pathogenic *DMD* variants is sometimes rather difficult, mainly due to complex structural variants (SVs) and deep intronic splice-altering variants. We performed genomic long-read whole *DMD* gene sequencing in a boy with asymptomatic hyper-creatine kinase-emia who remained genetically undiagnosed after standard genetic testing, dystrophin protein and *DMD* mRNA studies, and genomic short-read whole *DMD* gene sequencing. We successfully identified a novel pathogenic SV in *DMD* intron 1 *via* long-read sequencing. The deep intronic SV consists of a long interspersed nuclear element-1 (LINE-1) insertion/non-tandem duplication rearrangement causing partial exonization of the LINE-1, establishing a genetic diagnosis of Becker muscular dystrophy. Our study expands the genetic spectrum of dystrophinopathies and highlights the significant role of disease-causing LINE-1 insertions in monogenic diseases.

## Introduction

Dystrophinopathies, caused by pathogenic *DMD* variants and characterized by primary involvement in skeletal and/or cardiac muscle fibers, are among the most common inherited muscular dystrophies and mainly affect male patients (Birnkrant et al., 2018). Becker muscular dystrophy (BMD) and its two allelic forms (X-linked dilated cardiomyopathy and Duchenne muscular dystrophy) are the three major phenotypes of dystrophinopathies (Muntoni, Torelli, & Ferlini, 2003). The perplexing genetic spectrum of dystrophinopathies, as a result of the high genetic complexity of the *DMD* gene, includes both large-scale copy number variation or structural variants (SVs) and small pathogenic *DMD* variants, which can occur in coding and/or non-coding regions (Neri et al., 2020; Xie et al., 2020; Waddell et al., 2021; Xie et al., 2021). Most of the pathogenic *DMD* variants can be identified by DNA-based standard genetic testing for dystrophinopathies, consisting of multiplex ligation-dependent probe amplification (MLPA) and short-read sequencing of 79 exons and adjacent intronic regions of *DMD* (Fratter et al., 2020; Neri et al., 2020; Xie et al., 2021). However, standard genetic testing cannot identify some atypical pathogenic *DMD* variants, including complex SVs (Xie et al., 2020; Gonçalves et al., 2021; Waddell et al., 2021), deep intronic *DMD* variants (Gonçalves et al., 2017; Waddell et al., 2021; Xie et al., 2021), and synonymous variants which cause abnormal splicing of *DMD* pre-mRNA and result in abnormal dystrophin protein (Juan-Mateu et al., 2013). The detection and construction of these atypical pathogenic *DMD* variants require the application of various DNA- and RNA-based genetic testing techniques and *in silico* splicing analysis (Xie et al., 2021).

Herein, we performed standard genetic testing in a boy with a highly suspected BMD based on his clinical and pathological characteristics, which failed to detect pathogenic variant(s) in him. In order to discover potential deep intronic splice-altering variants in *DMD*, muscle biopsy-based dystrophin protein and *DMD* mRNA studies, as well as genomic short-read whole *DMD* gene sequencing, were performed on him. We successfully identified a novel deep intronic SV in *DMD via* genomic long-read whole *DMD* gene sequencing of the patient. The novel pathogenic SV missed by short-read whole *DMD* gene sequencing is a ∼6-kb-long interspersed nuclear element-1 (LINE-1) insertion/non-tandem duplication rearrangement, causing partial exonization of the LINE-1 in addition to the normal splicing of *DMD*, which ultimately established a genetic diagnosis of BMD in the patient.

## Methods and results

### Clinical and pathological features

**FIGURE 1:**
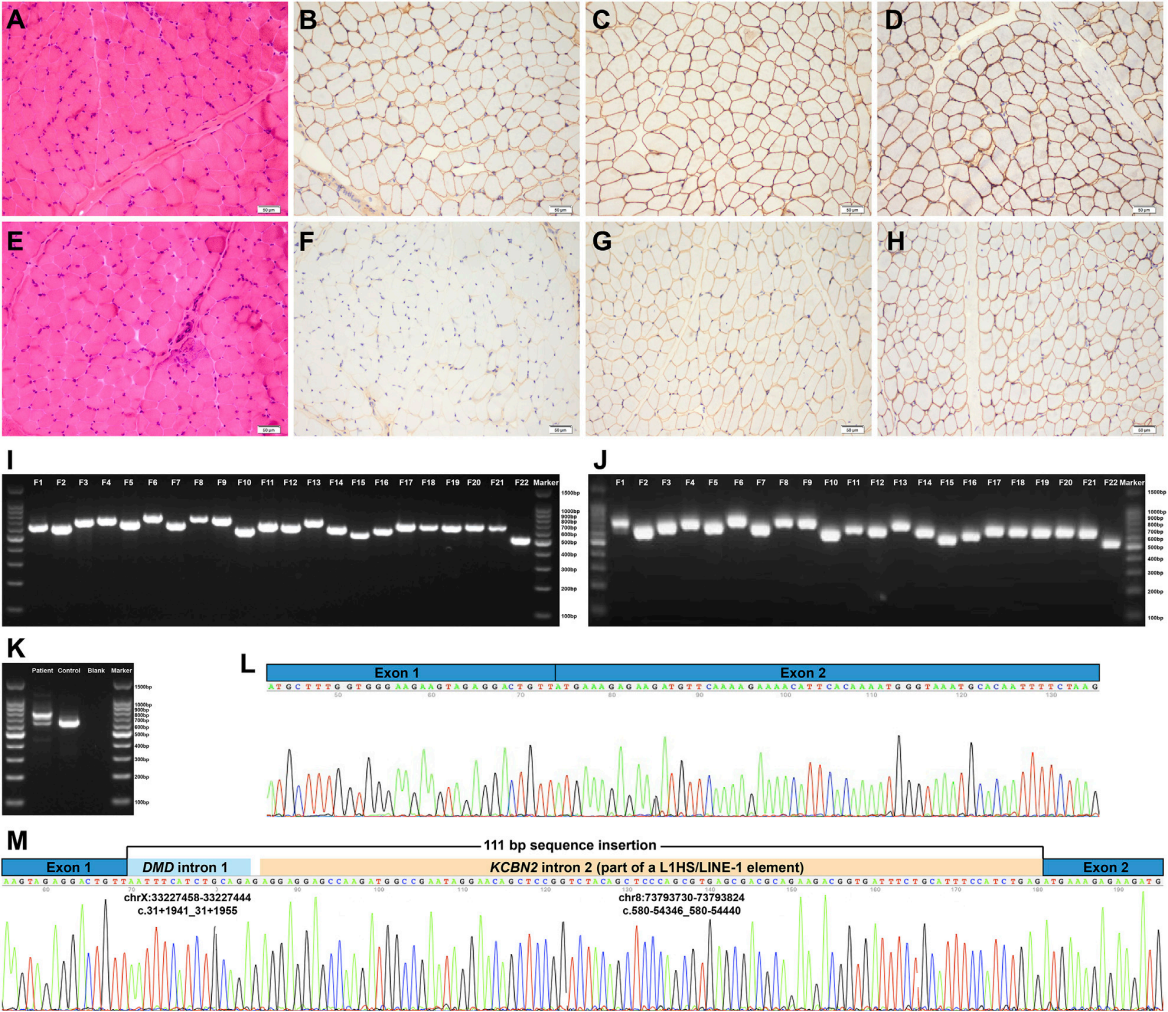
Pathologic features and *DMD* mRNA studies of the patient. **(A–D)** Normal control showing no pathological changes and positive expression of dystrophin-N, dystrophin-C, and dystrophin-R (200× magnification). **(E)** Hematoxylin–eosin staining revealed myopathic changes in the patient. **(F–H)** Immunohistochemical staining showing severe reduction of dystrophin-N, slight reduction of dystrophin-C, and positive expression of dystrophin-R. **(I)** Gel electrophoresis analysis of the amplified cDNA fragments confirmed that the 22 overlapping cDNA fragments were successfully amplified in the normal control I and the patient J. **(J)** Two different bands were found in F1, indicating the aberrant *DMD* transcripts. **(K)** Further RT-PCR analysis of the aberrant *DMD* transcripts revealed that the upper band was longer than the normal band, whereas the lower band was the same size as the normal band, that is, the wild-type transcript. **(L)** and **(M)** Sanger sequencing of the aberrant *DMD* transcripts revealed a 111-bp sequence insertion between *DMD* exons 1 and 2 in addition to the normal splicing of *DMD* exons 1 to 2. RT-PCR, reverse transcription–polymerase chain reaction; F, fragment.

The patient enrolled in the study is a 3.5-year-old boy with an asymptomatic hyper-creatine kinase-emia phenotype. He presented to Peking University First Hospital Neuromuscular Center at the age of 3.5 years because of an incidental finding of hyper-creatine kinase-emia. His serum creatine kinase was markedly elevated in every test (range 2,897–4,717 IU/L; normal 25–195 IU/L). No one else in his family had hyper-creatine kinase-emia. He had no delayed motor milestones and no exercise-induced muscle pain or cramps. Physical examination confirmed that he had mild calf hypertrophy and no muscle weakness, which was further validated by his muscle MRI examinations, showing no muscle fatty infiltration of the pelvis and thigh muscles. His muscle biopsy revealed myopathic changes, including several clusters of necrotic and regenerating muscle fibers; a few hypertrophic, atrophic, and hypercontracted muscle fibers; and a small number of internal nuclei (Figure 1E). The immunohistochemical staining showed a severe reduction of dystrophin-N, a slight reduction of dystrophin-C, and a positive expression of dystrophin-R (Figures 1F–H), which established a highly suspected diagnosis of BMD in the patient.

### Standard genetic testing and *DMD* mRNA analysis

We performed standard genetic testing for dystrophinopathies based on his suspected diagnosis of BMD, including MLPA-analysis of exonic deletion/duplication in *DMD* (copy number variations) and a short-read sequencing panel for inherited neuromuscular disorders (Xie et al., 2021). However, the standard genetic testing did not detect any disease-causing variants. Hence, we performed muscle-derived *DMD* mRNA analysis to identify potential abnormal *DMD* transcripts.

**FIGURE 2:**
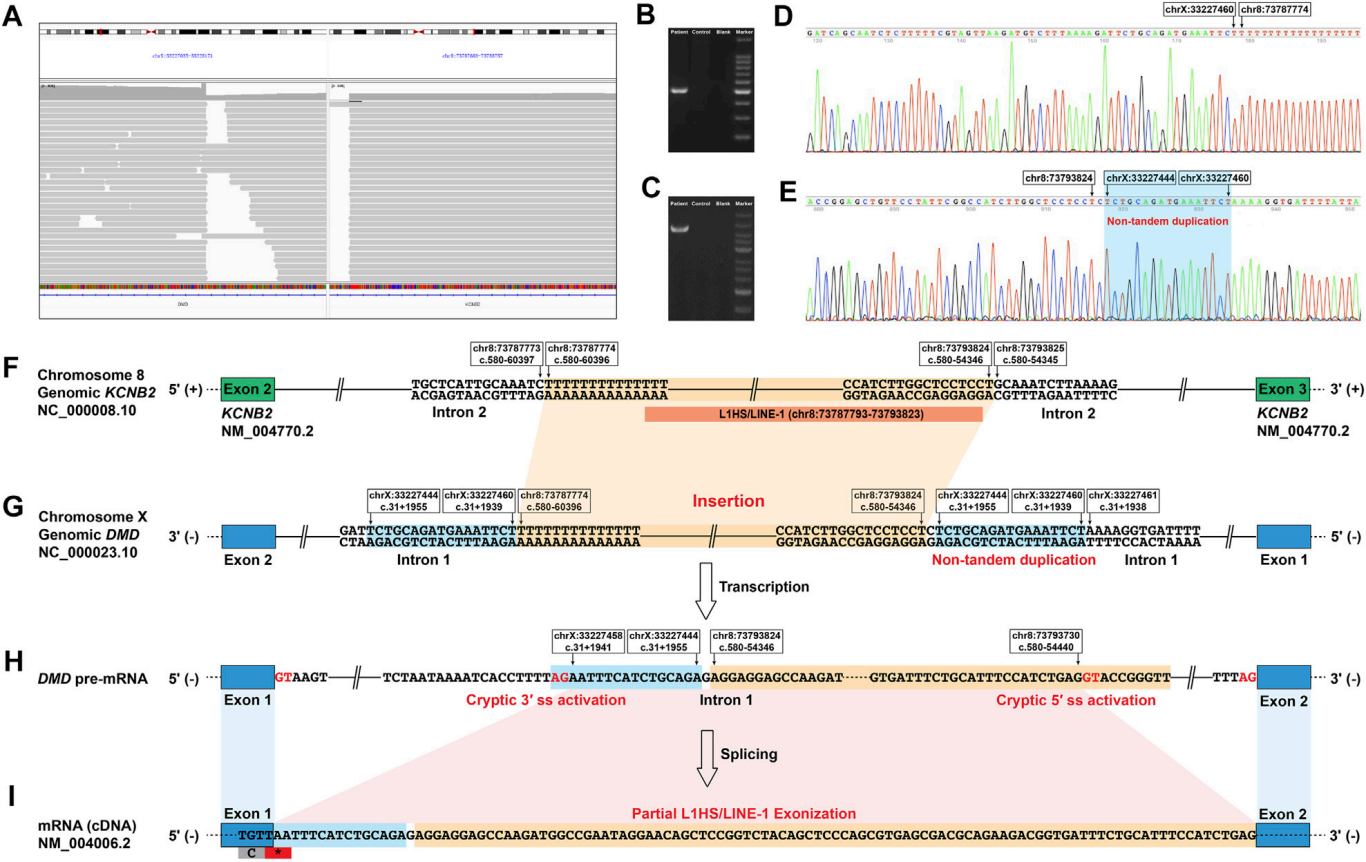
Identification of a novel deep intronic structural variant in *DMD* and its aberrant splicing effect on *DMD* pre-mRNA. **(A)** The IGV screenshot of long-read whole *DMD* gene sequencing of the patient indicated an insertion of a ∼6-kb sequence from chromosome 8 in the deep intronic region of *DMD* intron 1. Successful PCR amplification of the genomic 3′ **(B)** and 5′ **(C)** breakpoint regions using primers designed for the deep intronic *DMD* variant detected by the long-read whole *DMD* gene sequencing. Successful Sanger sequencing of the genomic 3′ **(D)** and 5′ **(E)** breakpoint regions confirmed the exact sequences of the breakpoint regions. **(F**–**I)** Schematic diagram of the abnormal *DMD* splicing event caused by the deep intronic structural variant in the patient. **(F)** and **(G)** The structural variant located in the deep intronic region of *DMD* intron 1, insertion of a 6,051-bp sequence originating from a deep intronic region in *KCNB2* intron 2 flanked by a non-tandem duplication of a 17-bp sequence originating from *DMD* intron 1. Most of the inserted sequence was annotated as an L1HS element belonging to the LINE-1 family. **(H)** The structural variant activated a cryptic 5′ ss in the LINE-1 element and a cryptic 3′ ss in the deep intronic region of *DMD* intron 1. **(I)** The cryptic 5′ ss and 3′ ss activation resulted in the 111-bp sequence insertion between *DMD* exons 1 and 2, consisting of a 15-bp sequence of *DMD* intron 1, a single guanine nucleotide, and the partial exonization of LINE-1 (95 bp). IGV, Integrative Genomics Viewer; L1 or LINE-1, long interspersed nuclear element-1; 5′ ss, donor splice site; 3′ ss, acceptor splice site; *, stop codon.

We extracted total mRNA from the remaining muscle biopsy sample and amplified 22 overlapping cDNA fragments of the full-length *DMD* mRNA (NM_004006.2, the primary full-length transcript in skeletal muscle) using the reverse transcription-polymerase chain reaction (RT-PCR) approach (Xie et al., 2021). Gel electrophoresis analysis of the 22 amplified cDNA fragments revealed aberrant splicing transcripts in the first cDNA fragment, showing two different bands; the upper band was longer than the normal band, whereas the lower band was the same size as the normal band, that is, the wild-type transcript (Figures 1J,K). Sanger sequencing of the aberrant *DMD* transcripts revealed a 111-bp sequence insertion between *DMD* exons 1 and 2 in addition to the normal splicing of *DMD* exons 1 to 2 (Figures 1L and M). The Human BLAT Search tool was adopted to search genomic sequences (GRCh37/hg19) that were homologous to the aberrant 111-bp sequence insertion. The homology search confirmed that the aberrant insertion consisted of a 15-bp sequence originating from *DMD* intron 1 (NM_004006.2:c.31+1941_31+1955), a single guanine nucleotide, and a 95-bp sequence homologous to a deep intronic region in *KCBN2* intron 2 (NM_004770.2:c.580-54346_580-54440; Figure 1M). The 95-bp sequence was part of an L1HS/LINE-1 element annotated by the RepeatMasker Web Server with default conditions (http://www.repeatmasker.org/cgi-bin/WEBRepeatMasker), that is, the partial exonization of LINE-1. According to the Human Genome Variation Society nomenclature (den Dunnen et al., 2016), the aberrant insertion transcript was described as follows: NM_004006.2:r.[=,31_32ins[NM_004006.2:r.31+1941_31+1955;g;NM_004770.2:r.580-54346_580-54440]]. The aberrant insertion transcript encoded a premature termination codon (NP_003997.1:p.[=,Tyr11*]; Figure 2I), which was targeted for degradation by nonsense-mediated decay. The aberrant insertion transcript and the normally spliced *DMD* transcript resulted in the decreased expression of dystrophin protein (Figures 1F–H).

### Genomic short- and long-read whole *DMD* gene sequencing

Genomic DNA was isolated from the peripheral blood of the patient, and short-read whole *DMD* gene sequencing was done as previously described (Xie et al., 2021) to detect possible variant(s) that could cause the aberrant insertion transcript. However, no possible aberrant splicing-causing variants were detected. Thus, we performed genomic long-read whole *DMD* gene sequencing (Xie et al., 2021) using the Nanopore PromethION (Oxford Nanopore, Oxford, United Kingdom) sequencer (average read depth 306X; average read length 3942 bp) to detect possible SVs that missed by the short-read whole *DMD* gene sequencing. Ninety-two reads with an average length of 4,362 bp indicating a ∼6-kb insertion originating from chromosome 8 in a deep intronic region of *DMD* intron 1 was identified. Exact sequences of the breakpoint regions were confirmed by Sanger sequencing of the genomic 3′ (Figures 2B,D) and 5′ (Figures 2C,E) breakpoint regions using primers designed for the deep intronic SV in *DMD* according to the genetic information provided by the 92 reads. The deep intronic SV in *DMD* intron 1 was an insertion/non-tandem duplication rearrangement, an insertion of a 6,051-bp sequence originating from a deep intronic region in *KCNB2* intron 2 (chr8:73787774-73793824) flanked by a non-tandem duplication of 17 bp sequence originating from *DMD* intron 1 (chrX:33227444-33227460), which was finally described as follows (Figures 2F,G): NC_000023.10:g.33227460_33227461ins[NC_000008.10:g.73787774_73793824; C;NC_000023.10:g.33227444_33227460]; NM_004006.2:c.31+1955_31+1956ins[G; NM_004770.2:c.580-54346_c.580-60396; NM_004006.2:c.31+1939_31+1955]. Most of the inserted sequence (chr8:73787793-73793823) was annotated as an L1HS/LINE-1 element (Figure 2F). The deep intronic SV identified in this study is classified as a novel pathogenic *DMD* variant according to the standard guidelines (Richards et al., 2015), which is not reported in the literature and absent from population and disease-specific databases, including the Genome Aggregation Database, Database of Genomic Variants, ClinVar, and Leiden Open Variation Database.

The novel deep intronic SV activated a cryptic donor splice site [GAG|GTACCG with a Human Splicing Finder score (Desmet et al., 2009) of 78.55 and a Maximum Entropy score (Yeo & Burge, 2004) of 8.5] in the LINE-1 element and a cryptic acceptor splice site (ATCACCTTTTAG|AA with a Human Splicing Finder score of 80.69 and a Maximum Entropy score of 6.31) in the deep intronic region of *DMD* intron 1, resulting in the aberrant splicing of *DMD* pre-mRNA (Figures 2H,I).

## Discussion

As the largest gene identified in the human genome, the *DMD* gene with a genomic size of over 2.5 Mb has numerous constitutive exons and very long introns. Common fragile sites and transposable elements, including DNA transposons and retrotransposons that can induce large and complex genomic rearrangements, are not uncommon in the vast intronic sequences of *DMD* (Muntoni et al., 2003). The accurate genetic counseling of dystrophinopathies relies primarily on the identification of underlying disease-causing *DMD* variants in the affected patients and the determination of the carrier status in the family (Kubota et al., 2022). The precise identification of pathogenic *DMD* variants is important to ensure that correct candidates are subjected to the different emerging personalized therapies.

In addition to exonic *DMD* variants, which directly cause alterations in dystrophin protein sequences and constitute the majority of pathogenic *DMD* variants, deep intronic variants that can cause aberrant *DMD* splicing and subsequent defects in dystrophin protein are increasingly found in patients with dystrophinopathies (Xie et al., 2021). The precise identification of deep intronic *DMD* variants demands various DNA- and RNA-based genetic testing approaches due to the genetic complexity of *DMD*. In the present study, we performed *DMD* mRNA studies in a boy with asymptomatic hyper-creatine kinase-emia who remained without pathogenic variants after standard genetic testing and detected an aberrant *DMD* splicing event. Genomic short-read whole *DMD* gene sequencing was then performed to detect possible variant(s) causing the aberrant splicing event. As no potential splice-altering variants were detected through short-read whole *DMD* gene sequencing, genomic long-read whole *DMD* gene sequencing was performed in the patient, which detected a novel pathogenic SV in the deep intronic region of *DMD* intron 1. The novel deep intronic SV is a ∼6-kb LINE-1 insertion/non-tandem duplication rearrangement identified through long-read sequencing, which further indicates that short reads are not enough to cover variants involving repetitive elements or large-scale SVs (Xie et al., 2020). The pathogenic *DMD* variants involving repetitive elements, such as the novel genomic SV causing partial exonization of the LINE-1 identified in our patient, might be a significant cause of genetically unsolved dystrophinopathies. This study highlights the importance of long-read sequencing in the detection and construction of pathogenic *DMD* variants involving repetitive elements. Precise detection and construction of deep intronic splice-altering variants in *DMD* have potential implications for the genetic therapy targeting normal dystrophin expression.

Long interspersed nuclear elements, typically spanning about 6 kb in length, belong to the retrotransposons, which cover about 17% of the human genome (Kim, Lee, & Han, 2012). LINE-1s, which are difficult to detect with the short-read sequencing technique such as the novel *DMD* SV identified in our patient, can cause inherited diseases through various pathogenic mechanisms, including *de novo* LINE-1 insertions, LINE-1 insertion-mediated deletions, and genomic rearrangements (Kim et al., 2012). To our knowledge, only eight patients with dystrophinopathy have been reported to be caused by pathogenic *DMD* variants involving LINE-1s. One of the eight patients is caused by a LINE-1 insertion in the 5′ untranslated region of *DMD*, causing instability of the mature *DMD* mRNA (Yoshida, Nakamura, Yazaki, Ikeda, & Takeda, 1998), one is caused by a LINE-1-meditated large-scale SV in *DMD* causing multiple exons-skipping (Xie et al., 2020), two are caused by deep intronic LINE-1s insertions causing partial exonization of LINE-1s (Gonçalves et al., 2017; Xie et al., 2021), and four are caused by exonic LINE-1 insertions causing exon-skipping (Narita et al., 1993; Holmes, Dombroski, Krebs, Boehm, & Kazazian, 1994; Musova et al., 2006; Awano et al., 2010). Our study is the third to report on a deep intronic LINE-1 insertion causing partial exonization of the LINE-1, expanding the spectrum of pathogenic *DMD* variants and highlighting the significant role of disease-causing LINE-1 insertions in monogenic diseases.

## Data Availability

The structural variant data presented in the study have been submitted to Leiden Open Variation Database, accession number Variant #0000872856.
